# Examining the Role of Sublingual Atropine for the Treatment of Sialorrhea in Patients with Neurodevelopmental Disabilities: A Retrospective Review

**DOI:** 10.3390/jcm12165238

**Published:** 2023-08-11

**Authors:** Kayla Durkin Petkus, Garey Noritz, Laurie Glader

**Affiliations:** 1 Division of Complex Care, Nationwide Children’s Hospital, Columbus, OH 43205, USA; garey.noritz@nationwidechildrens.org (G.N.); laurie.glader@nationwidechildrens.org (L.G.); 2 Department of Pharmacy, Nationwide Children’s Hospital, Columbus, OH 43205, USA

**Keywords:** atropine, cerebral palsy, sialorrhea, pediatric, drooling, sublingual, developmental disorders, neurodevelopmental disorders

## Abstract

Sialorrhea is common in children with neurodevelopmental disabilities (NDD) and is reported in >40% of children with cerebral palsy (CP). It causes a range of complications, including significant respiratory morbidity. This single-center retrospective chart review aims to document sublingual atropine (SLA) utilization to guide further study in establishing its role in secretion management for children with NDD. A chart review was completed for patients with NDD ≤ 22 years of age treated with SLA at a free-standing children’s hospital between 1 January 2016 and 1 June 2021. Descriptive statistics were generated to summarize findings. In total, 190 patients were identified, of which 178 met inclusion criteria. The average starting dose for SLA was 1.5 mg/day, or 0.09 mg/kg/day when adjusted for patient weight. Eighty-nine (50%) patients were prescribed SLA first line for secretion management while 85 (48%) patients tried glycopyrrolate prior to SLA. SLA was used after salivary Botox, ablation, and/or surgery in 16 (9%) patients. This study investigates SLA as a potential pharmacologic agent to treat sialorrhea in children with NDD. We identify a range of prescribing patterns regarding dosing, schedule, and place in therapy, highlighting the need for further evidence to support and guide its safe and efficacious use.

## 1. Introduction

Sialorrhea, or excessive drooling, is a common problem for children with neurodevelopmental disabilities (NDD), particularly those with motor disabilities. Prevalence among children with cerebral palsy (CP), the most common motor NDD, is over 40% [1]. The term CP describes “a group of permanent disorders of the development of movement and posture, causing activity limitation, that are attributed to nonprogressive disturbances that occurred in the developing fetal or infant brain. The motor disorders of cerebral palsy are often accompanied by disturbances of sensation, perception, cognition, communication, and behavior, by epilepsy, and by secondary musculoskeletal problems [2].” In the US, the prevalence of CP is estimated between 1.5 and 4 per 1000 live births [3]. Other childhood motor disabilities in which sialorrhea is common include the muscular dystrophies, spinal muscular atrophy, sequelae of traumatic brain injury, and neurodegenerative conditions such as Rett syndrome and the leukodystrophies. Children with craniofacial abnormalities, airway abnormalities, or tracheostomies may also have significant sialorrhea. Finally, drooling is a common symptom amongst people with intellectual disability disorder, and it is a reasonably common side effect of medication commonly used in children with neurologic disorders, including benzodiazepines and atypical neuroleptics. 

Sialorrhea can manifest anteriorly, as spillage of saliva over the lips, or posteriorly, with pooling of secretions in the oropharynx. Anterior sialorrhea tends to be associated with skin breakdown and irritation, odor, and can impact social relationships and self-esteem. Posterior sialorrhea can have serious medical sequelae, such as difficulty with airway clearance, chronic aspiration, and recurrent pneumonia [4]. Pulmonary issues are a leading cause of morbidity in children with complex medical conditions, accounting for 29% of hospitalizations in the population [5,6,7]. Respiratory disease is also the most common cause of death for both children and adults with CP, underscoring the importance of providing effective interventions, such as sialorrhea reduction, to curb pulmonary complications [8,9]. 

Both anterior and posterior sialorrhea affect the health and quality of life of children and their families. As such, numerous strategies were developed in an effort to reduce symptoms. Common approaches to management range from least to most invasive. These typically include behavioral interventions, pharmacologic treatments, targeted salivary gland injections with botulinum toxin, and finally, surgeries, which include salivary duct ligation, re-routing, or gland excision [10]. Ultrasound-guided salivary gland ablation using ethanol is an emerging intervention [11]. Treatment appears to be effective with all agents, but studies are heterogeneous, with generally low-level evidence and inconsistency in outcome measures [10,11,12,13,14,15].

Behavioral treatments for sialorrhea are based on principles of behavior modification and include reinforcement, prompting, self-management, extinction, overcorrection, instruction, and fading. These are discussed in a recent systematic review, which concluded that “Low-level evidence suggests behavioural interventions may be useful for treatment of drooling in children with neurodisability [15]”.

Among pharmacologic agents used to treat sialorrhea, anticholinergics are the most common. Glycopyrrolate, scopolamine, trihexyphenidyl, and benztropine are the most studied agents [10,16,17,18,19,20,21]. More recently, sublingually administered atropine (SLA) appeared in the literature [22]. As a newer agent to be utilized in this context, atropine’s recommended dosing and place in treatment is still being established.

Atropine competitively blocks acetylcholine from binding to muscarinic receptors in the central nervous system, smooth muscle, and secretory glands. Submucosal glands are innervated by parasympathetic neurons and predominantly have M1 and M3 muscarinic receptors. Activation of these receptors by acetyl choline is blocked by atropine, inhibiting secretions from the nose, mouth, pharynx, and bronchi. Ultimately, this leads to drying of the mucus membranes in these areas [23,24].

Injectable forms (intramuscular (IM), intravenous (IV), and subcutaneous (SubQ)) of atropine have an approved indication to inhibit salivation and secretions in the preoperative/intraoperative setting. Recommended dosing for inhibition of salivation in infants, children, and adolescents is 0.02 mg/kg/dose administered IM, IV, or SubQ. Doses may be repeated every 4 to 6 h as needed for secretions. The maximum total dose for infants and children less than 12 years of age is 1 mg per procedure, while the maximum total dose for children greater than or equal to 12 years of age is 2 mg per procedure [25].

A study completed by Volz-Zang and colleagues assessed how oral versus intramuscular administration of atropine affected the heart rate and salivary flow in seven healthy adults. They found that a 0.03 mg/kg dose of atropine administered orally demonstrated an 84.3% maximum reduction in salivary flow. The orally administered dose did not cause a significant increase in heart rate, especially when compared to intramuscular administration of the medication. They attributed their findings to low absorption of the oral dose and to lower vagal tone in the salivary glands compared to the heart [26]. Schwartz et al. additionally demonstrated systemic bioavailability of SLA at 60%, which is achieved via an intravenous route in healthy adults [27]. Whether there is a direct local effect on the salivary glands remains unclear. Available studies suggest a more limited side effect profile for SLA compared to alternative agents, such as glycopyrrolate and scopolamine, which have well-documented potential effects of constipation, urinary retention, and behavioral changes, among others [28,29,30,31]. The reduced systemic absorption, the report to date of minimal adverse effects, and the rapidity of a drying effect from the sublingual administration of atropine make it an attractive option for treating children with sialorrhea.

Most reports of using SLA in the literature are in palliative settings for adults, case reports involving pediatric patients, or for the treatment of pharmacologically induced sialorrhea [32,33,34]. In a pediatric case report, dosing of SLA for sialorrhea was reported as atropine 0.5% ophthalmic drop, given as one drop (0.25 mg) sublingually every six hours as needed. Based on the need for suctioning, it was estimated that the onset of action was between 15 and 30 min and the duration of action was approximately 4 h [33].

Two prospective studies evaluated efficacy in pediatric patients with NDD [30,31]. Dias et al. performed an uncontrolled open clinical trial of 33 children with cerebral palsy, 25 of whom completed the study. Significant reduction in drooling was reported as reflected in the validated parent reported Drooling Impact Scale across all but two variables [30,35]. Norderyd et al. evaluated 19 children in a prospective single system design where participants served as their own controls, 11 of whom completed the study. Significant reduction in drooling was measured using the Visual Analogue Scale, another parent reported instrument [31]. Both studies found tolerable adverse effects [30,31]. Dias’ study identified fever and flushing (1), irritability (1), flushing and irritability (1), and flushing and angioedema (1) in 4 of 33 patients [30]. Norderyd reported that excessive dry mouth occurred most frequently (7), followed by difficulty with voiding, constipation, and behavioral changes (3 in each case). All side effects disappeared with cessation of treatment [31]. Azapagasi et al. conducted a retrospective chart review of 25 hospitalized children receiving SLA for 7 days, 20 of whom had outcome data available. Significant reduction in the Teacher Drooling Scale, a 5-point reporting scale, was reported over a two-day period (*p* < 0.001). Side effects were not reported [36].

The literature for SLA is thus sparse and the evidence is generally of a low level. Although it paints an optimistic view of the potential for SLA to be used in the treatment of sialorrhea in children and young adults with NDD, further study is clearly warranted. The current study was undertaken to describe use of SLA, including dosing regimens and place in therapy, in an effort to glean patterns of real-world use and to inform potential dosing regimens for prospective study.

## 2. Materials and Methods

This study is a retrospective electronic chart review evaluating SLA use for sialorrhea within a free-standing children’s hospital. Data were extracted from our electronic health record for patients who received an outpatient prescription order for atropine 1% ophthalmic solution, atropine 0.5% ophthalmic solution, and/or atropine 0.5% oral solution (compounded at our institution once the 0.5% ophthalmic solution was discontinued by manufacturers in 2014) for sublingual administration between 1 January 2016 and 1 June 2021. Patients with NDD of childhood, an umbrella term for conditions associated with neurologic impairment that impacts physical, cognitive, linguistic, and behavioral development and function, were eligible. Inclusion criteria included being 22 years of age or younger at therapy initiation and being treated with SLA either alone or in conjunction with other anti-cholinergic therapies for secretion management. Exclusion criteria included use of SLA for other indications, absence of NDD, age > 22 years at treatment initiation, incomplete data, and/or patients with protected chart information.

Patient information including date of birth, sex, and unifying diagnosis was collected to describe our population. For the first and last SLA prescription listed in the patient’s chart, date of prescription, patient dosing weight, medication, dose, and frequency were collected. Doses were calculated based on product concentration and assuming the established criteria that 20 drops is equal to one milliliter of clear solution [37]. For daily dose and weight-based dose calculations, all “as needed” doses were considered to be given as scheduled, and doses were rounded to the maximum dose if a dosing range was given. Additional analysis was completed to assess if dosing differed depending on the patient’s age at the start of therapy (<1 year, 1–<3 year, 3–<12 year, and ≥12 year) to incorporate common age cut offs for dosing of another anticholinergic agent, glycopyrrolate, in other studies.

A manual chart review of all medication history was performed to identify whether the patient previously received alternative anticholinergic therapies for the treatment of sialorrhea (i.e., glycopyrrolate, scopolamine, trihexyphenidyl, and/or benztropine). Timing of when these agents were utilized for secretion management in relation to SLA therapy was assessed. A manual chart review was also completed to evaluate prior administration of salivary gland botulinum toxin injection, salivary gland surgery, and/or salivary gland ablation.

Descriptive statistics were generated to summarize our findings. Data are presented as means, ranges, and percentages. A two-tailed paired *t*-test was completed to assess significance of change from starting to final dose. This study was approved by the Institutional Review of Nationwide Children’s Hospital and a waiver of consent was granted.

## 3. Results

On initial chart review, 190 eligible patients were prescribed SLA for the treatment of sialorrhea. Seven patients were excluded due to missing or incomplete chart data, 3 patients were removed due to protected chart information, and 2 were removed due to the absence of a neurodevelopmental diagnosis. The remaining 178 patients were included in the analysis. Patient demographic information is included in Table 1. The average age at initiation of atropine was 7.8 years, and 96 patients were male (54%). Ninety-eight patients (55%) had a confirmed diagnosis of cerebral palsy (CP). It is quite possible that children with some of the diagnoses listed, such as brain injury or genetic disorder, met criteria for a diagnosis of CP but did not carry the diagnosis in their chart, resulting in an underestimate of this subpopulation. Almost half of the initial prescriptions (*n* = 88, 49.4%) were generated through the pulmonary service. Fifty-five prescriptions (30.9%) were written by the complex care division, which cares for children with medical complexity. The remaining prescriptions were written by various subspecialties, including neonatal services, neurology, acute care, physical medicine, aero-digestive, palliative care, and otorhinolaryngology, or at discharge from the general pediatric hospital service.

Dose and dosing frequency of SLA ranged greatly among patients [Table 2]. Doses ranged from 1 to 3 drops given at a time, and frequency ranged from every 4 to every 24 h, often accompanied by indications of “may increase up to X” for both dosing and scheduling. The most common starting frequency was written as twice daily (*n* = 63, 35.4%) and approximately one-third of the prescriptions were written for “as needed” (PRN) dosing. Final dosing strategies were also quite varied and open to adjustment.

The most common starting dose was atropine 1% drops, 2 total drops per day, regardless of age or weight at the start of therapy, and ranged up to 12 drops per day. Dosing by drop was converted to mg/day as well as mg/kg/day to allow for analysis of weight-based treatment strategies and for direct comparison to results of prior studies. It is important to note that prescriptions were not written in a weight-based manner, but rather reflected the practicality of using doses standardized by common concentration and drop volume. The difference between average starting and final mg/day was significantly different (1.5 mg vs. 1.8 mg, *p* ≤ 0.001), but when adjusted for patient weight, there was no significant difference based on mg/kg/day (*p* = 0.635). Upon examining the variance in mg/kg dosing between initial and final prescriptions for SLA, 128 patients (72%) had no change in dose, while 40 patients (22%) had an increase in dose. By comparison, mg/kg dosing remained consistent.

Initial and final sublingual atropine dosing, expressed as drops, are presented in this table and resemble the “real life” approach to dosing this medication. Please note that all doses provided as ranges were rounded up for the purpose of analysis.

We found that SLA dosing for sialorrhea management in this population was quite variable. For ease of analysis, a side-by-side table is provided [Table 3] to allow for comparison of prescribing patterns in this study with the three studies previously mentioned [30,31,36].

**Figure 1:**
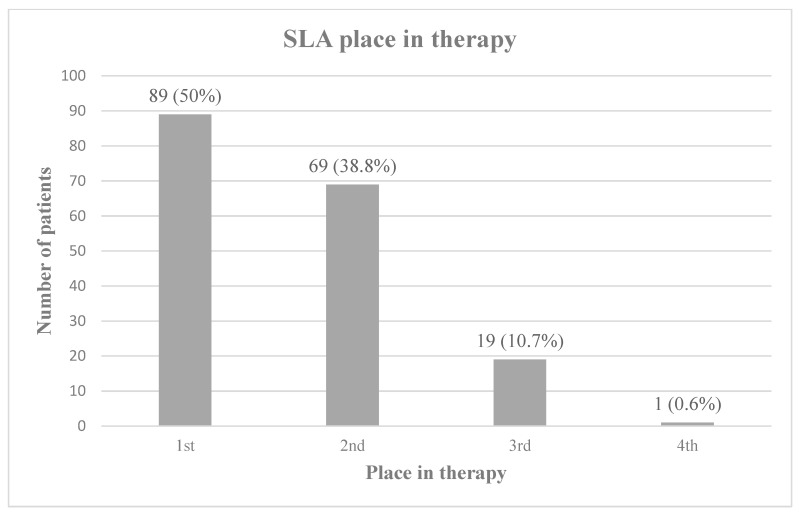
SLA place in therapy. SLA’s place in therapy relative to alternative anticholinergic medications, including glycopyrrolate, scopolamine, benztropine, and trihexyphenidyl. Percentages do not total 100 due to rounding.

When compared to other anticholinergic therapies used for sialorrhea, SLA was prescribed as a first or second line agent for the majority of patients in this study [Figure 1].

**Figure 2:**
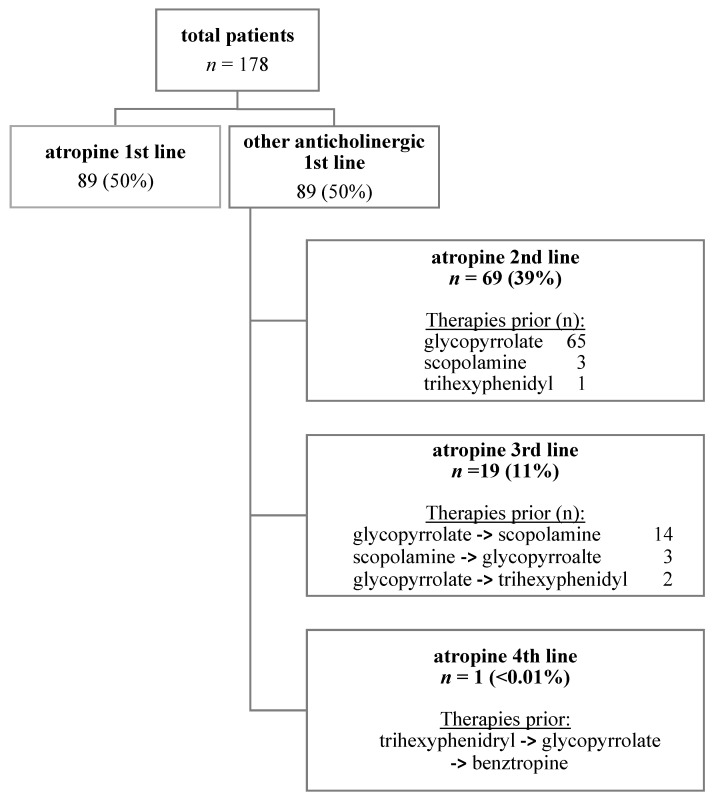
Pharmacologic sialorrhea treatment regimens in relation to SLA.

Eighty patients (44.9%) were on SLA in addition to at least one other anti-cholinergic therapy (atropine with glycopyrrolate, scopolamine, benztropine, and/or trihexyphenidyl) at some point during their course of treatment with atropine. Anticholinergic therapies trialed prior to atropine are summarized in Figure 2. Glycopyrrolate was used prior to SLA in 85 (47.8%) patients and scopolamine was used prior to atropine in 20 (11.2%).

Sixteen patients underwent more invasive therapies prior to the initiation of SLA. All patients who had medication data available in the chart (*n* = 13) trialed glycopyrrolate, while two patients trialed scopolamine in addition to glycopyrrolate prior to moving towards more invasive therapy options and, subsequently, onto SLA. Invasive treatments included: salivary gland ablation (*n* = 7 patients, 3.9%), submandibular salivary gland surgery (*n* = 4, 2.2%), botulinum toxin injection into the salivary glands (*n* = 3, 1.7%), salivary gland botulinum toxin followed by ablation (*n* = 1, 0.6%), and salivary gland surgery followed by ablation (*n* = 1, 0.6%).

## 4. Discussion

This study presents a single institution’s real-world practice in relation to already available literature in order to advance the breadth and depth of literature available regarding the use of SLA in the treatment of sialorrhea in children and young adults with NDD. Two previous prospective small cohort studies and one retrospective study suggest the potential efficacy of SLA for this purpose. Our study evaluated the current prescribing practice of SLA in children and young adults with NDD to understand the scope of need to establish prescribing guidance and safety profiles. We did not evaluate efficacy in drooling reduction, nor did we evaluate adverse events, as that information was not available through chart review in a standardized manner.

We found significant variability in actual practice among prescribers, and there was no institutional standardized dosing guidance. Initial doses ranged from one to three drops and frequencies ranged from as needed to six times daily. Although six times daily dosing was infrequent (*n* = 14, 7.9%), this same pattern was seen in the Azapagasi study [31]. The dosing range, in mg/day, documented in our study far exceeded that studied in the three previously mentioned cohorts [28,29,31]. However, our calculations assumed that all doses written for “as needed” were given at scheduled dosing intervals. Although this possibly falsely elevated upper limits of actual practice, the prescriptions were, importantly, written to allow for this possibility. Despite the wider range of dosing prescribed, most regimens were in keeping with the Dias and Norderyd studies [28,29]. More than 90% of regimens were written for 1–2 drops per dose of the atropine 1% solution (0.5 mg/drop), and >80% were written for up to TID dosing frequency. Overall, weight-based doses remained fairly stable from initial to final prescriptions, potentially reflecting the expected weight gain of growing children during the study period.

While it was not possible to assess for side effects in our study, those observed in other studies were reversible and of minimal impact [22,28,29]. Anticholinergic side effects, including urinary hesitancy or retention, constipation, flushing, and the desired effect of decreased saliva production, are the most commonly reported side effects from SLA in this setting. Some of the more serious potential side effects include arrhythmias, ataxia, and respiratory failure [20] and were not reported to date in this body of literature. The limited available data suggest that the dosing regimens explored thus far may be useful in defining a safe range for assessing efficacy and safety. Future studies will require monitoring for the full constellation of potential adverse effects.

Despite a lack of established safety or dosing guidance, SLA is a tool frequently used in the treatment of sialorrhea in children with NDD. At our institution, atropine was used as a first-line anti-cholinergic agent in patients 50% of the time, and second to other anti-cholinergic treatments 50% of the time, generally after glycopyrrolate, which is the only agent that carries an FDA approval for chronic drooling in children ages 3 to 16 years. Atropine in the dosage form described in this study is dispensed in a dropper bottle and is typically administered into the eyes. When prescribed for administration via the sublingual route, there is risk of misuse, accidental overdose, or toxicity. Prescribers, nursing staff, and consumers/patients should receive thorough education regarding SLA indication, use, administration, and side effects. It should also be recommended to store the dropper bottle in a child-proof container and keep out of reach of small children.

This descriptive medication use evaluation is a first step in examining actual SLA use in children and young adults with NDD. There are several limitations to this study. Our work reflects local prescribing habits at a single institution. However, SLA is used widely for this application without guidelines, and it is possible to surmise that similar variability in practice might exist more broadly. Based on the data available for this study, it was not possible to evaluate efficacy, side effects, adherence to therapy, or rationale for therapy cessation. Outside records were not reviewed to obtain previous exposure to anticholinergic agents, botulinum toxin, surgery, and/or ablation outside of our institution. Additionally, to capture the maximum potential dosing made by prescribers, our dosing may be overestimated as we rounded up on dosing intervals and assumed scheduled doses when prescriptions were written on an “as needed” basis.

## 5. Conclusions

Our study summarizes the data for SLA use within a single institution. It documents that SLA is commonly used to treat drooling in children and young adults with NDD and that the prescribing practices are highly variable. We identify a lack of consistency regarding dosing, schedule, and place in therapy for SLA use in this population. Importantly, it draws attention to the wide practice variation that often evolves in the absence of guidelines. Efficacy, dosing regimens and adverse events of SLA require additional exploration. Further rigorous prospective study is necessary to establish the safety and efficacy of SLA for the treatment of sialorrhea in children and young adults with NDD. We hope that this preliminary study will form the basis for a randomized control trial of SLA in comparison to a more established therapy, such as enteral glycopyrrolate.

## Figures and Tables

**Table 1:** Demographic information.

	Total *n* = 178 *n* (%)
Age in years at initiation of atropine	
Average (range)	7.8 (2 months–22 years)
<1 year	21 (11.8%)
1–<3 years	26 (14.6%)
3–<12 years	80 (44.9%)
>12 years	51 (28.7%)
Gender	
Male	96 (54%)
Female	82 (46%)
Underlying Diagnosis	
Cerebral Palsy	98 (55.1%)
Genetic/syndromic disorder	35 (19.7%)
Neuromuscular disease	17 (9.6%)
Neurodegenerative condition	11 (6.2%)
Brain injury	5 (2.8%)
Developmental disorder	4 (2.2%)
Multiple congenital anomalies	4 (2.2%)
Unknown/other	4 (2.2%)

**Table 2:** SLA dosing by drop.

	Initial *n* = 178 *n* (%)	Final *n* = 178 *n* (%)
Drops per dose		
Atropine 0.5%		
1 drop	9 (5%)	0 (0%)
2 drop	6 (3.4%)	1 (0.56%)
Atropine 1%		
1 drop	125 (70.2%)	126 (70.8%)
2 drop	36 (20.2%)	47 (26.4%)
3 drop	2 (1.12%)	3 (1.7%)
4 drop	0 (0%)	1 (0.56%)
Frequency of dosing		
QDay	44 (24.7%)	37 (20.8%)
BID	63 (35.4%)	60 (33.7%)
TID	38 (21.3%)	42 (23.6%)
4 times daily	19 (10.7%)	25 (14%)
6 times daily	14 (7.9%)	14 (7.9%)
Total drops per day		
Atropine 0.5%		
1 drop	2 (1.1%)	0 (0%)
2 drop	3 (1.337%)	0 (0%)
3 drop	1 (0.56%)	0 (0%)
4 drop	1 (0.56%)	1 (0.56%)
6 drop	5 (2.8%)	0 (0%)
12 drop	3 (1.7%)	0 (0%)
Atropine 1%		
1 drop	36 (20.2%)	31 (17.4%)
2 drop	51 (28.7%)	48 (27%)
3 drop	24 (13.5%)	28 (15.7%)
4 drop	25 (14%)	31 (17.4%)
6 drop	17 (9.6%)	21 (11.8%)
8 drop	7 (3.9%)	10 (5.6%)
9 drop	1 (0.56%)	1 (0.56%)
12 drop	2 (1.1%)	7 (3.9%)
Rx written as PRN	66 (37%)	79 (44.4%)

Abbreviations: QDay, once daily; BID, twice daily; TID, three times daily; and Rx, prescription.

**Table 3:** Comparison of available data. “Side-by-side” comparison of patient populations, products used, directions for use, and dosing data found in our study versus that available in the literature. Some calculations were completed to extrapolate directions/doses from their original form to ease comparison.

Study	Our Study	Dias et al. [30]	Norderyd et al. [31]	Azapagasi et al. [36]
Population	≤22 years with NDD (*n* = 178)	2-17 years with CP (*n* = 25)	5–18 years with disabilities (final study group *n* = 11)	PICU patients 3–78 months (*n* = 20, of whom 19/20 had a NDD)
Product	atropine 0.5% ophthalmic drop; atropine 1% ophthalmic drop; atropine 0.5% oral solution	atropine 0.5% ophthalmic drop	atropine 1% ophthalmic drop	atropine sulfate ampoule
Directions	Varied/retrospective observation, initial dosing	Give 1 drop SL TID at 6h intervals for patients 10–19 kg Give 2 drops SL TID at 6h intervals for patients ≥20 kg	After 3 weeks of no treatment, Give 1 drop QDay for 4 weeks followed by 1 drop BID for 4 weeks	0.02 mg/kg/dose 4-6 times daily for 7 days Minimum dose was 0.25 mg, Maximum dose was 0.03 mg/kg (per author)
Drops/dose	1–3 drops	10–19 kg: 1 drop ≥20 kg: 2 drop	1 drop	N/A
Frequency	QDay-6 times daily	TID	QDay-BID	4–6 times daily
mg/day	0.25–6 mg/day Average: 1.5 mg/day	10–19 kg: 0.75 mg/day * ≥20 kg: 1.5 mg/day *	0.5–1 mg/day ^†^	1 mg/day ^‡^-range unknown
mg/kg/day	0.01–0.49 mg/kg/day Average: 0.091 mg/kg/day	10–19 kg: 0.04–0.075 mg/kg/day * ≥20 kg: ≤0.075 mg/kg/day *	NA	0.08–0.18 mg/kg/day ^§^

* Calculations were made by extrapolating on the reported data to allow comparison in discussion with the primary author. ^†^ Calculation was made by extrapolating on the reported data to allow comparison. ^‡^ Dose based on reported 0.25 mg minimum dose given 4 times daily. ^§^ Dose based on giving 0.02 mg/kg/dose 4 times daily to 0.03 mg/kg/dose 6 times daily to determine minimum and maximum dosing strategies. Abbreviations: NDD, Neurodevelopmental Disability; CP, Cerebral Palsy; PICU, Pediatric Intensive Care Unit; SL, sublingual; TID, three times daily; h, hour; kg, kilogram, QDay, once daily; BID, twice daily.

## Data Availability

The data presented in this study are not publicly available due to privacy restrictions. Questions relating to the data may be addressed by contacting the corresponding author.

## Abbreviations

NDD, Neurodevelopmental Disability; CP, Cerebral Palsy; SLA, sublingual atropine; IM, intramuscular; IV, intravenous; SubQ, subcutaneous; Rx, prescription; PRN, as needed; QDay, once daily; BID, twice daily; TID, three times daily.
